# Principal component analysis of blood microRNA datasets facilitates diagnosis of diverse diseases

**DOI:** 10.1371/journal.pone.0234185

**Published:** 2020-06-05

**Authors:** Stacy L. Sell, Steven G. Widen, Donald S. Prough, Helen L. Hellmich

**Affiliations:** 1 Department of Anesthesiology, The University of Texas Medical Branch at Galveston, Galveston, Texas, United States of America; 2 Department of Biochemistry and Molecular Biology, The University of Texas Medical Branch at Galveston, Galveston, Texas, United States of America; Gustave Roussy, FRANCE

## Abstract

Early, ideally pre-symptomatic, recognition of common diseases (e.g., heart disease, cancer, diabetes, Alzheimer’s disease) facilitates early treatment or lifestyle modifications, such as diet and exercise. Sensitive, specific identification of diseases using blood samples would facilitate early recognition. We explored the potential of disease identification in high dimensional blood microRNA (miRNA) datasets using a powerful data reduction method: principal component analysis (PCA). Using Qlucore Omics Explorer (QOE), a dynamic, interactive visualization-guided bioinformatics program with a built-in statistical platform, we analyzed publicly available blood miRNA datasets from the Gene Expression Omnibus (GEO) maintained at the National Center for Biotechnology Information at the National Institutes of Health (NIH). The miRNA expression profiles were generated from real time PCR arrays, microarrays or next generation sequencing of biologic materials (e.g., blood, serum or blood components such as platelets). PCA identified the top three principal components that distinguished cohorts of patients with specific diseases (e.g., heart disease, stroke, hypertension, sepsis, diabetes, specific types of cancer, HIV, hemophilia, subtypes of meningitis, multiple sclerosis, amyotrophic lateral sclerosis, Alzheimer’s disease, mild cognitive impairment, aging, and autism), from healthy subjects. Literature searches verified the functional relevance of the discriminating miRNAs. Our goal is to assemble PCA and heatmap analyses of existing and future blood miRNA datasets into a clinical reference database to facilitate the diagnosis of diseases using routine blood draws.

## Introduction

Many devastating diseases, including heart disease, cancer, diabetes, Alzheimer’s disease (AD) and other dementias, are partially preventable through lifestyle interventions such as diet and physical activity [1]. Patients with, or at risk for, many of these diseases would benefit from earlier diagnosis, especially if therapies or lifestyle modifications are available that improve outcome (S1 Reference). Because blood samples are easily accessible and can be repeatedly sampled, detection and assessment of circulating biomarkers would allow an individualized approach to early disease management [2]. Regulatory microRNAs (miRNAs), which are stable in blood and other circulating biofluids, represent potential non-invasive, disease-specific biomarkers [3].

In 2014, NIH director Francis Collins described the potential value of archived datasets in publicly accessible databases and suggested that mining existing ‘Big Data’ (genetic, phenotypic and clinical) could identify new predictive markers of disease risk [4]. One such database includes thousands of blood miRNA datasets maintained at the National Center for Biotechnology Information’s (NCBI) Gene Expression Omnibus (GEO) database. However, mining these complex datasets has typically required expertise in statistics, mathematics, bioinformatics, and machine learning techniques [5].

One solution to the mining of big datasets is to use established data reduction techniques, such as principal component analysis (PCA), that effectively reduce a large set of variables into a smaller, easier-to-analyze set without losing the meaningful information contained in the large set [6]. In our studies of humans with Traumatic Brain Injury (TBI), we found that PCA of blood miRNA profiles clearly distinguished patients with TBI from uninjured subjects, even TBI patients that suffered as long as 32 years previously [7]. This demonstrated that circulating miRNAs could serve as stable biomarkers of human disease. Here, we extend those observations using a commercially available bioinformatics program, Qlucore Omics Explorer (QOE), which executes dynamic, interactive PCA. Using QOE, we found that a broad spectrum of human diseases are characterized by significant alterations in circulating miRNAs in blood or blood components. Literature searches validated the functional relevance of discriminating miRNA markers in the pathology of each disease. Here, we present a series of bioinformatic analyses demonstrating that one of the obstacles to personalized medicine, the management and analysis of big datasets, can be addressed using PCA and heatmap analyses of miRNA expression in blood samples. We also show evidence that the discriminating miRNA variables identified in these analyses can serve as diagnostic and prognostic biomarkers of specific diseases.

## Methods

### Principal component analysis of miRNA datasets using Qlucore Omics Explore

Principal component analysis is a way to identify strong patterns in large, complex datasets. This widely used data reduction technique captures the essential information in high-dimensional data by identifying a few principal components that account for most of the variability in the dataset; PCA finds the maximum variance in each variable (how far each value in the dataset is from the mean) and then projects the variance of these many variables into a smaller, easier-to-analyze set of linearly uncorrelated principal components. For example, observing a group of people from a substantial distance, differences in height, body habitus and hair length would permit generally accurate identification of men and women. In such a group, these variables (height, body habitus and hair length) would represent the first, second and third principal components, respectively.

**Fig 1:**
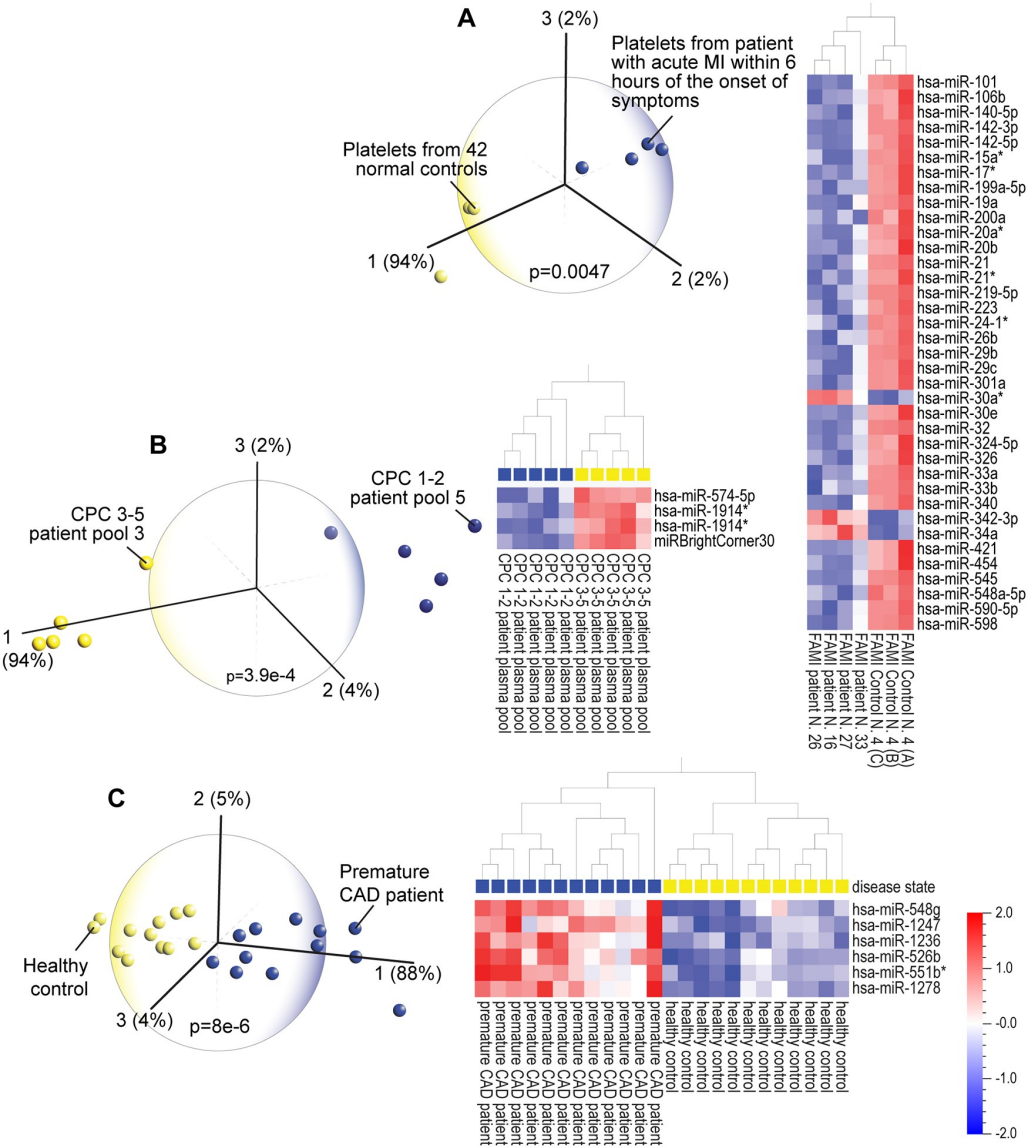
(A) Principal component analysis (PCA) and hierarchical clustering heatmap of miRNA expression in platelets of patients affected by first acute myocardial infarction (FAMI) and in platelets from normal controls (GEO accession # GSE24548). The 37 miRNAs that distinguish the two groups account for 98% of the variance in the dataset. (B) PCA and hierarchical clustering heatmap of patients with favorable (1–2) vs poor (3–5) cerebral performance category (CPC) neurological outcome after cardiac arrest (GSE34643) shows that four miRNAs, that account for 98% of the variance in the dataset, can clearly distinguish the patients with poor or favorable outcomes. (C) PCA and hierarchical clustering heatmap of premature coronary artery disease (CAD are a young age) vs healthy controls (GSE28858) shows that six miRNA variables distinguish the CAD from healthy control groups. These six miRNAs represent 97% of the variance in the miRNA dataset.

Qlucore Omics Explorer (Qlucore, Lund, Sweden) is a data analysis and data mining software tool built on state-of-the-art mathematical and statistical methods (a general linear statistical model based on R), that combines speed and advanced analytics for interactive exploration and instant visualization of high-dimensional data. The user interface instantly responds to the adjustment of statistical parameters to represent the three principal components that are most responsible for the variance in a dataset. The display is intuitive and easily understandable, regardless of one’s depth of familiarity with data analytics or statistics. The sum of the three principal components in a PCA plot provides valuable information about the significance of the discriminating data, for instance in the Fig 1A hierarchical clustering heatmap, the top three principal components are represented by 37 miRNA variables that collectively represent 98% of the variance in the entire dataset; that is, these 37 miRNAs together are sufficient to distinguish the patients from the healthy subjects.

The workflow for downloading and analyzing blood miRNA datasets in QOE is shown in S1 Table. The program assembles the data matrix, calculates the means, subtracts the means from the data matrix, calculates the covariance matrix which captures the information about the spread of the data, and calculates the Eigen vectors and Eigen values of the covariance matrix. The first principal component is the Eigen vector corresponding to the largest Eigen value, the second principal component is the Eigen vector corresponding to the second largest Eigen value, etc. As statistical parameters are adjusted to appropriate significance levels, the PCA plots are instantly generated and updated. Hierarchical clustering is an algorithm that groups similar variables into clusters represented by a dendrogram (tree diagram). In QOE, hierarchical clustering heatmaps are instantly updated with their corresponding PCA plots. Following QOE’s identification of patients, we examined the discriminating variables (significant miRNAs) shown on the heatmaps that result from the PCA plots and performed literature searches on the discriminating miRNA variables to determine their functional relevance.

## Results

Data are grouped by related themes for ease of presentation. The seven figures represent: 1) heart disease, 2) hemodynamic diseases, e.g. stroke, hypertension and sepsis-induced acute kidney injury (AKI), 3) diabetes, 4) cancer subtypes, 5) diseases for which the discriminating miRNA variables in the heatmap analyses reveal pro-survival mechanisms, 6) two nervous system disorders with similar phenotypes and 7) brain disorders. All datasets in this study are identified by unique GEO accession numbers which are provided in the figure legends. Each GEO submission file contain a brief summary of the experimental paradigm and if available, a link to the published report. We observed that many GEO submissions are not published. The complete data files are publicly available and can be downloaded into bioinformatic programs or saved in Excel for further study.

### PCA and hierarchical clustering heatmap analyses identified patients with or at risk for heart disease

We performed PCA so that the top three principal components and the resulting discriminating miRNA variables displayed on the hierarchical clustering heatmaps represented 80–100% of the variance in each dataset. For example, PCA of blood miRNA datasets identified patients diagnosed with first acute myocardial infarction (FAMI; Fig 1A), patients with favorable (1–2) vs poor (3–5) cerebral performance category (CPC) neurological outcome after cardiac arrest (Fig 1B) and patients with coronary artery disease (CAD; Fig 1C). In FAMI, 98% of the variance in the entire dataset is represented by three principal components consisting of 37 discriminating miRNA variables. In CPC outcome after cardiac arrest, four miRNAs represent 100% of the variance. In CAD, six miRNAs represent 97% of the variance. PCA of miRNA profiles also clearly identified patients with unstable angina pectoris (S1 Fig).

Investigation of the functional roles of the discriminating miRNAs from the hierarchical clustering heatmaps provided key mechanistic insights; the majority of the miRNAs have roles in inflammation and immune regulation. Across all three datasets, miRNAs that showed elevated expression in healthy control subjects are associated with good cardiac function [8–10] or limited cardiac dysfunction after myocardial infarction [11]. Increased miRNAs in acute myocardial infarction (AMI) have been identified as potential biomarkers of heart disease [12] or modulators of heart function such as miR-200a [13] and miR-24 [14].

The functional roles of the identified miRNAs from Fig 1 correspond with current understanding of mechanisms underlying heart disease and other diseases with shared pathophysiology. Six examples are: 1) reduced circulating levels of miR-199 and miR-223 are associated with heart failure and atherosclerosis [15]; 2) miR-219-5p, which is elevated in healthy subjects and downregulated in AMI patients, promotes recovery from spinal cord injury by inhibiting inflammation and oxidative stress [16]; 3) miRNAs such as miR-223 limit inflammation in other diseases such as cancer [17]; 4) elevated miR-26b is associated with attenuated microglial-mediated inflammation [18]; 5) miR-29 has a role in reducing inflammation and fibrosis after liver injury [19]; and, 6) lower expression of heart disease-associated miRNAs, such as miR-20, is found in other inflammatory diseases such as rheumatoid arthritis [20]. An interesting association between heart disease and hypertension involves members of the miR-17 family. MiR-17 is downregulated in AMI and associated with CAD [21], as well as kidney disease with hypertension [22]. Upregulated expression of miR-574-5p is found in patients after cardiac arrest and also is associated with increased severity of CAD [23]. CAD-related miRNAs are associated with other forms of heart disease as well as other diseases; miR-1247 is associated with cardiac fibrosis and cell death [24] and miR-1236 [25], miR-548 [26] and miR-551b [27] are linked to inflammation and immune activation. Furthermore, deficiencies of specific miRNAs may result in pathology. Deficiency of miR-33 in mice (also decreased in FAMI) promotes obesity, insulin resistance and hyperlipidemia [28].

In some instances, the association of the miRNA with the underlying molecular function has been directly described. One example is miR-101 which is upregulated in SH-Sy5y cells in response to increased expression of TNF-α leading to increased inflammation [29]. This study by Han et al. showed that myocardial infarction associated transcript 2 (Mirt2) suppresses miR-101 indirectly through suppression of TNF-α, resulting in anti-inflammatory effects [29], thus demonstrating a direct role for miR-101 in inflammation. In our study of FAMI we found miR-101 to be highly expressed in the healthy subjects (Fig 1A), highlighting the complexity of the roles of miRNAs which can mediate both dysfunctional (i.e. inflammatory) and protective (i.e. anti-inflammatory) cellular responses. In this case, we infer that the protective effects of low miR-101 expression in FAMI patients are insufficient to counter the pro-inflammatory effects of the other miRNA changes.

Our analysis revealed more suggestive evidence that changes in miRNA expression associated with disease or healthy subjects can be protective responses. Both miR-30a (high in FAMI) and miR-326 (high in healthy subjects) resolve inflammation by targeting and reducing the expression of inflammatory mediators such as IL-1α [30, 31]. High levels of miR-342-3p (higher in FAMI) suppress inflammation and lipid uptake in human macrophages [32]. Therapeutic inhibition of miR-34a (decreased in healthy controls) leads to atherosclerosis regression and reverses diet-induced metabolic disorders [33]. Interestingly the anti-inflammatory effects of drugs have been shown to be mediated, in part, by miRNAs, e.g. dexmedetomidine reduces neuroinflammation via upregulation of miR-340 [34]. The cardioprotective effects of the flavonoid nobiletin are attributed to its reduction of lipid accumulation and secretion of proinflammatory cytokines via its upregulation of miR-590 [35].

### PCA accurately detects stroke, hypertension, and sepsis-induced acute kidney injury

**Fig 2:**
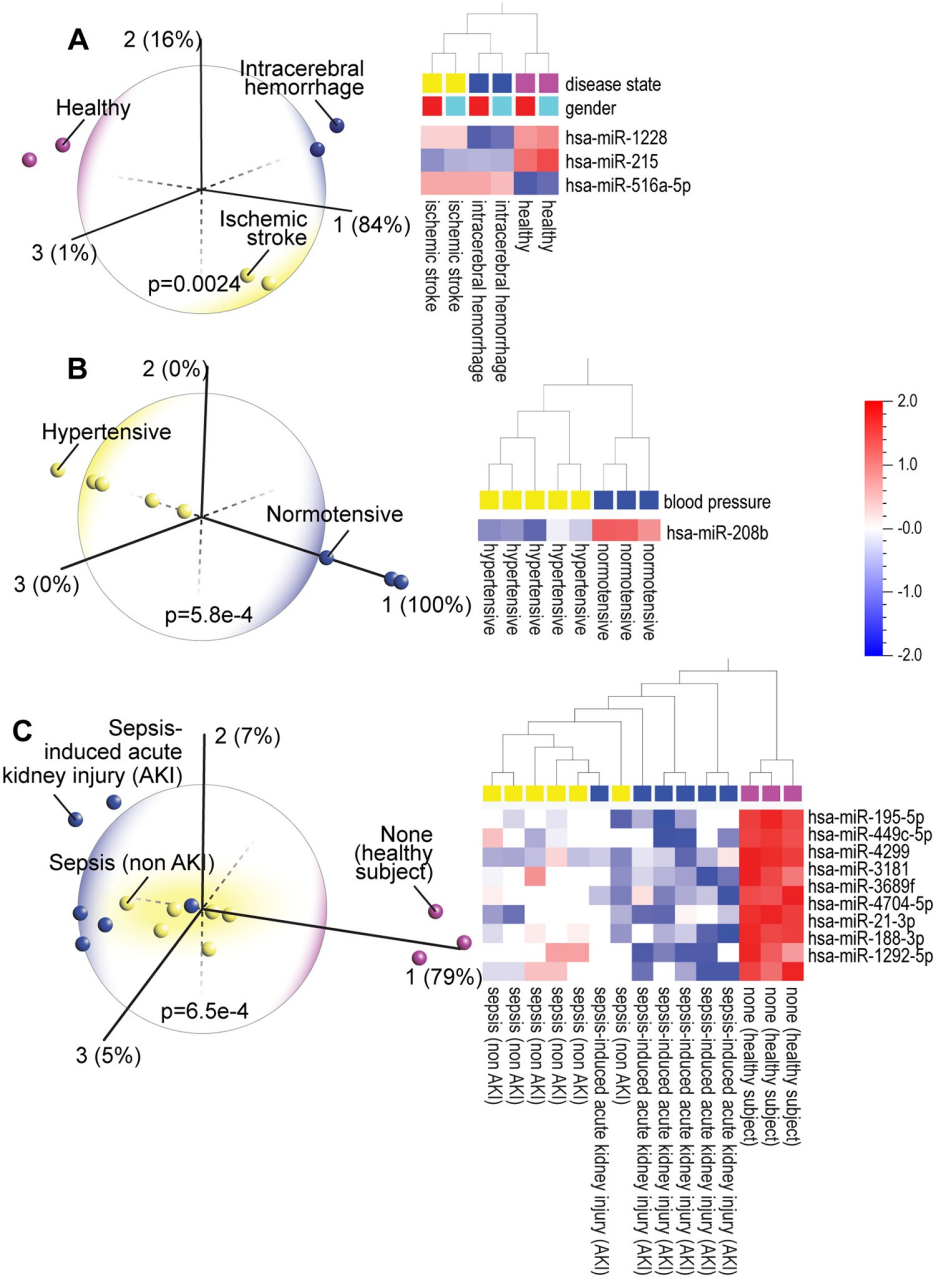
(A) Principal component analysis (PCA) and hierarchical clustering heatmap of pooled blood plasma miRNA expression profiles in intracerebral hemorrhage and ischemic stroke patients and in healthy controls (GSE43618) shows that differential expression of three miRNAs, similarly expressed in males (blue) and females (red), can distinguish the three groups from each other. (B) PCA and hierarchical clustering heatmap of renal medulla miRNA expression in hypertensive and normotensive patients (GSE28344) shows that one miRNA, miR-208b) is sufficient to discriminate between the two groups. (C) PCA and hierarchical clustering heatmap shows that the majority of sepsis-induced acute kidney injury (AKI) patients (GSE94717) can be distinguished from sepsis patients and from healthy subjects.

The expression of only three miRNAs, similarly expressed in men and women, is sufficient to discriminate patients with ischemic stroke from those with hemorrhagic stroke and from healthy subjects [Fig 2A; each column represents four pooled blood samples from males (blue) or females (red)]. Two miRNAs (miR-1228 and miR-215) that help distinguish between the intracerebral hemorrhage and ischemic stroke patients and each disease from healthy subjects, are linked to stroke [36]. Increased expression of miR-215 was shown to be neuroprotective against ischemic injury [37]. Hierarchical clustering heatmap analysis showed that miR-215 is upregulated in healthy control subjects while it is downregulated in both the ischemic and hemorrhagic stroke groups. Because high blood pressure increases risk of stroke and heart disease, we performed PCA on a dataset comparing hypertensive (HT) and normotensive (NT) patients. A single miRNA, miR-208b, which clearly distinguished HT from NT (Fig 2B), is highly expressed in the NT group, and is known to target Bax, a gene involved in apoptosis. Bax protects against hypoxia-induced apoptosis, and thus cardiovascular disease [38]. In data comparing sepsis and sepsis-induced AKI (Fig 2C), three miRNAs, miR-195 [39] miR-449c [40] and miR-3181 [41], which discriminate between sepsis and sepsis-induced AKI, are linked to regulation of inflammation and oxidative stress. Increased levels of miR-21, which is highly expressed in the healthy subjects, protect against sepsis-induced AKI [42] as well as oxidative stress [43] and miR-188, which is also high in healthy subjects, inhibits inflammation and atherosclerosis [44] and is implicated in cardiac remodeling [45]. Dysregulated levels of miR-4299 are also found in other diseases including amyotrophic lateral sclerosis (ALS) [46].

### PCA and hierarchical clustering analyses provide mechanistic insight into diabetes and heart disease and dementia risk in diabetic patients

**Fig 3:**
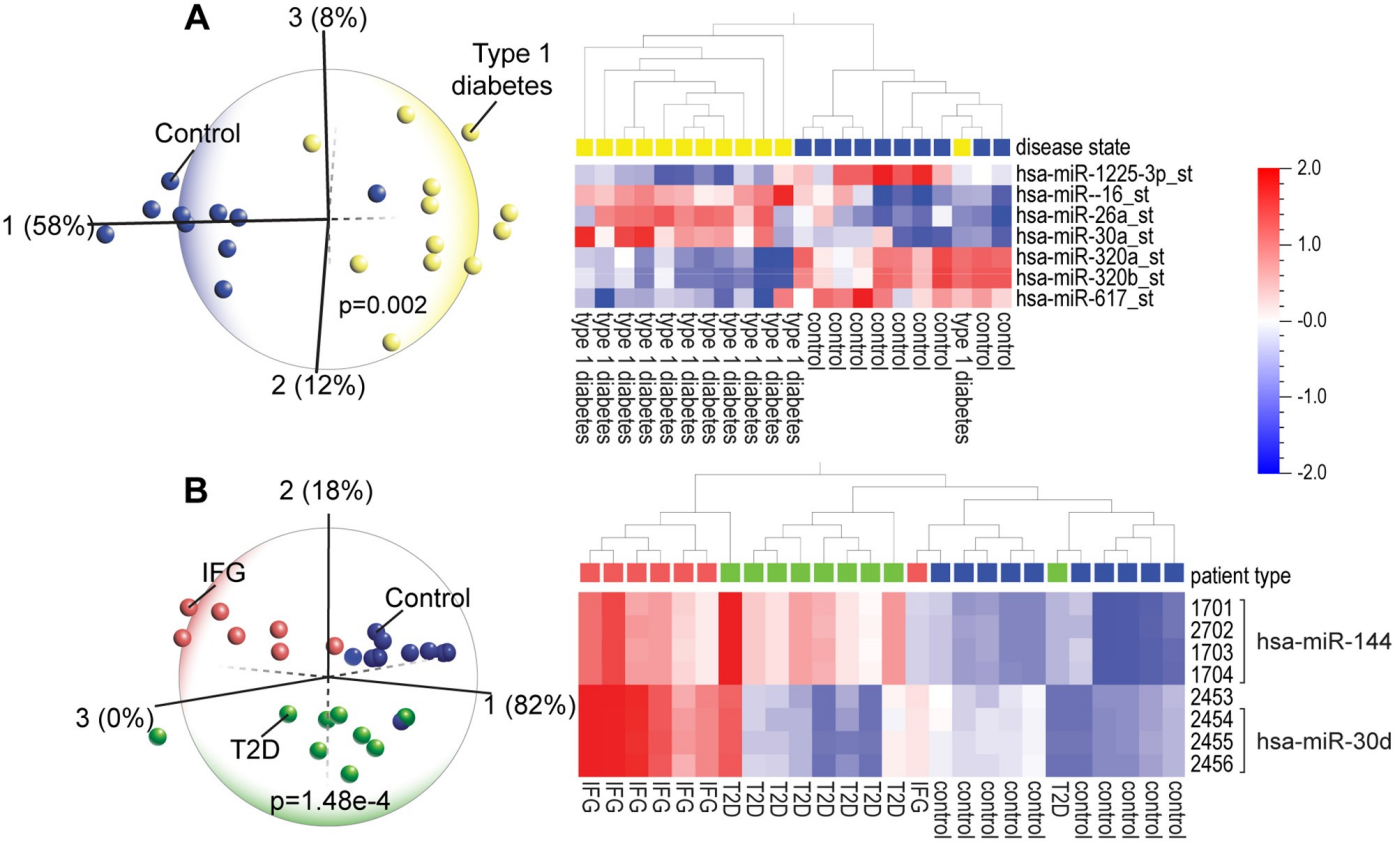
(A) Principal component analysis (PCA) and hierarchical clustering heatmap of peripheral blood mononuclear cell miRNA expression profiles in type 1 diabetes patients and normal controls (GSE55099) shows that a set of seven miRNAs can identify the majority of the diabetes patients. (B) PCA and hierarchical clustering heatmap of blood miRNA expression in patients with impaired fasting glucose (IFG), type 2 diabetes (T2D) and healthy controls (GSE21321) shows that differential expression of two miRNAs (miR-144 and miR-30d) can distinguish the three groups from each other. In the heatmap, each miRNA is represented by four probes.

Death of pancreatic beta cells is a major factor in the pathogenesis of type 1 diabetes (T1D) [47]. We found that miR-1225, is upregulated in healthy subjects and downregulated in TID patients (Fig 3A). Increased expression of miR-1225 was shown to inhibit apoptosis of pancreatic cancer cells, potentially allowing cell proliferation and promoting cancer [48]. Thus, decreased expression of miR-1225 in T1D suggests increased apoptosis of pancreatic cells, potentially promoting the development of diabetes, by increasing destruction of beta cells. Furthermore, high levels of miR-16, which are seen in T1D patients, are also found in women diagnosed with gestational diabetes mellitus [49] and are involved in insulin sensitivity [50]. Other discriminating miRNAs such as miR-26a are linked to autoimmune dysfunction in diabetes [51]. High levels of miR-26a and miR-30a found in T1D have functional roles in diabetic nephropathy [52], while miR-320 regulates glucose-induced gene expression in diabetes [53].

In a comparison of blood miRNA profiles among men with impaired fasting glucose (IFG), type 2 diabetes (T2D) and healthy subjects, we found that the IFG profile is exactly opposite that of healthy profiles and the T2D profile shows an intermediate miRNA signature (Fig 3B). The functional roles of the discriminating miRNAs in T2D are concordant with their biological relevance. For instance, mir-144, which distinguishes IFG and T2D from heathy subjects, impairs insulin signaling [54] and is linked to cognitive dysfunction. Given this context, it is relevant that T2D patients are at increased risk for AD and vascular dementia, and that metformin, a diabetes drug, protects against AD [55]. The two miRNAs, miR-144 and miR-30, that discriminate among the IFG, T2D and control groups, are the same miRNAs that identify the AD and mild cognitive impairment (MCI) patients (see Fig 7). Together, this information suggests two important ideas: 1) the dysregulation of a common set of miRNAs is evidence that there is a mechanistic link between the diseases; and 2) miRNAs may be useful blood biomarkers for diagnosis as well as monitoring the course of a disease and the response to treatment.

### PCA and hierarchical clustering analyses identified distinct cancer subtypes

**Fig 4:**
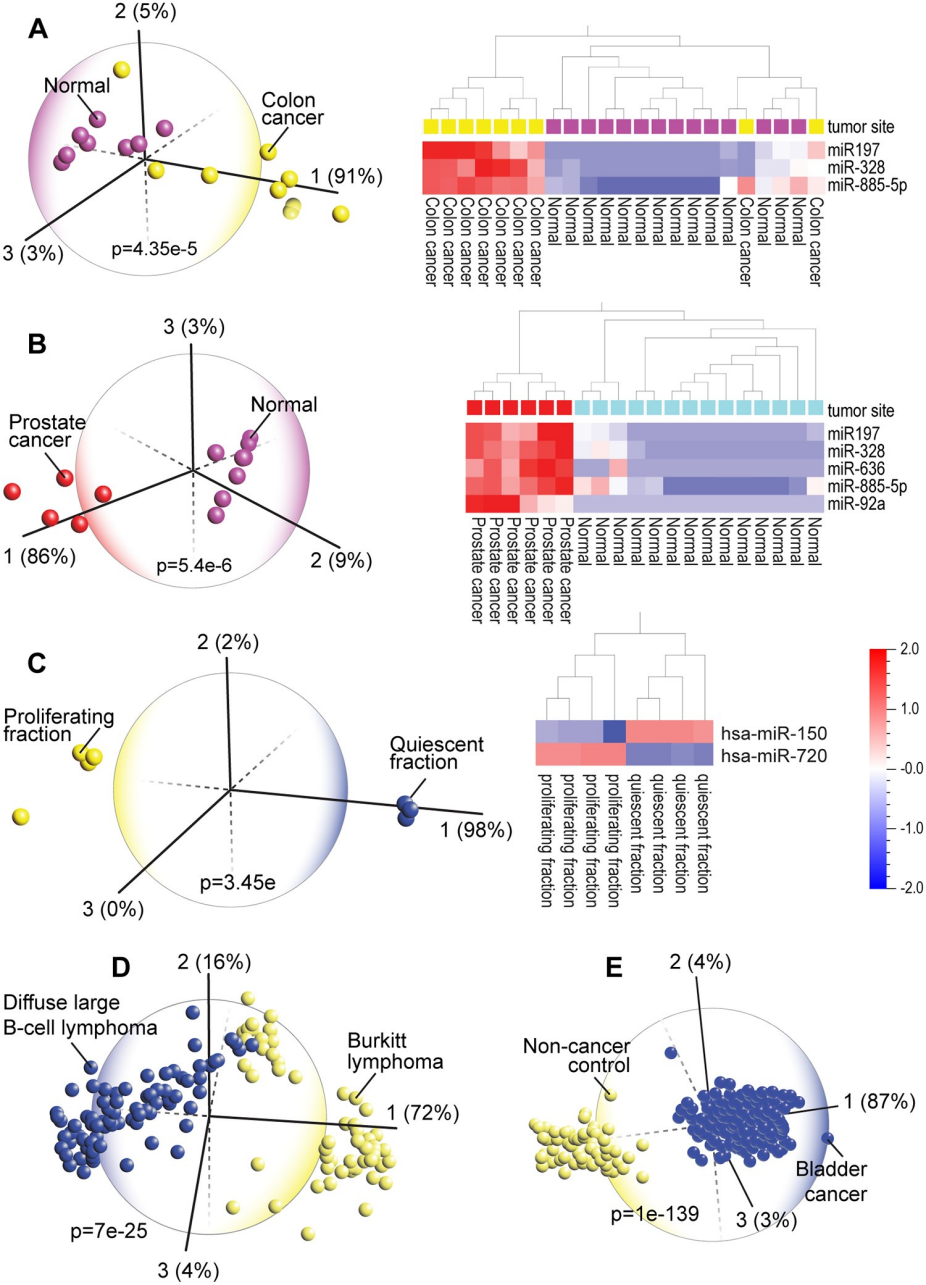
Principal component analysis (PCA) and hierarchical clustering heatmap of blood serum miRNA expression profiles from various types of cancer patients (GSE16512) shows that patients with colon (A) and prostate (B) cancers, which have been shown to be part of the same tumor spectrum, can be discriminated form normal controls and that these related cancers display similar differential expression of a common set of three miRNAs (miR-197, miR-328 and miR-885-5p). (C) PCA and heatmap analysis clearly distinguished between quiescent and proliferating chronic lymphocytic leukemia (GSE53235). Only two miRNAs are sufficient to distinguish the proliferating from quiescent fractions in chronic lymphocytic leukemia B-cells. (D). Analysis of miRNA levels in tissues of Burkitt lymphoma and Diffuse large B-cell lymphoma patients (GSE22420)- these are challenging to distinguish based on heatmap alone- shows that PCA can clearly identify the majority of patients in these cancers. (E) PCA can clearly distinguish patients in other cancers such as bladder cancer (GSE113486).

Cancer is the second leading cause of death (1 in 6 deaths) globally [56]. Earlier diagnosis increases the chance of survival. Circulating miRNAs can identify different types of cancer [57]. In men, analysis of blood miRNA profiles of two common cancers, colon and prostate cancer, showed that patients with each cancer type can be clearly distinguished from healthy subjects. In fact, these two cancers can be distinguished from each other by two miRNAs (miR-636 and miR-92a) but they also display similar expression of a set of miRNAs (miR-197, miR-328, miR-885-5p) that are also found dysregulated in other cancers (Fig 4A and 4B). Previous studies showed that miR-197 is dysregulated in colorectal [58] and prostate cancer [59]. Inhibition of miR-328, which is minimally expressed n healthy patients, impairs proliferation of cancer stem cells and inhibits metastasis [60] and miR-636 is a marker of pancreatic cancer [61]. MiR-885, which is highly expressed in both prostate and colon cancers, is also linked to liver cancer [62]. Many of the miR-92a family members may serve as diagnostic biomarkers of a variety of cancers [63].

The central role of miRNAs in human disease was first demonstrated in chronic lymphocytic leukemia, a cancer of blood-forming tissues [64]. Our analysis showed that PCA can discriminate between quiescent and proliferating chronic lymphocytic leukemia (Fig 4C). High levels of miR-720, which has been shown to promote the migratory and invasive phenotype of triple negative breast cancer cells [65] and low levels of miR-150, which is linked to aggressive B-cell malignancies, clearly distinguish the proliferating fraction of chronic lymphocytic leukemia B-cells [66]. Differentiating cancer subtypes is often a diagnostic challenge. For example, it is difficult to differentiate between Burkitt lymphoma (BL) and diffuse large B-cell lymphoma (DLBCL) [67]. Nonetheless, using PCA we confirmed a previous study that showed that these two subtypes are distinct via miRNA profiling [68]. The complex hierarchical clustering heatmaps of these and other cancers (S2, S3 and S4 Figs) illustrate the difficulty of a differential diagnosis based purely on a heatmap alone. However, PCA enabled a clear identification of the majority of patients diagnosed with either subtype (Fig 4D) as well as bladder cancer (Fig 4E).

### PCA and heatmap analyses shed light on pro-survival mechanisms

**Fig 5:**
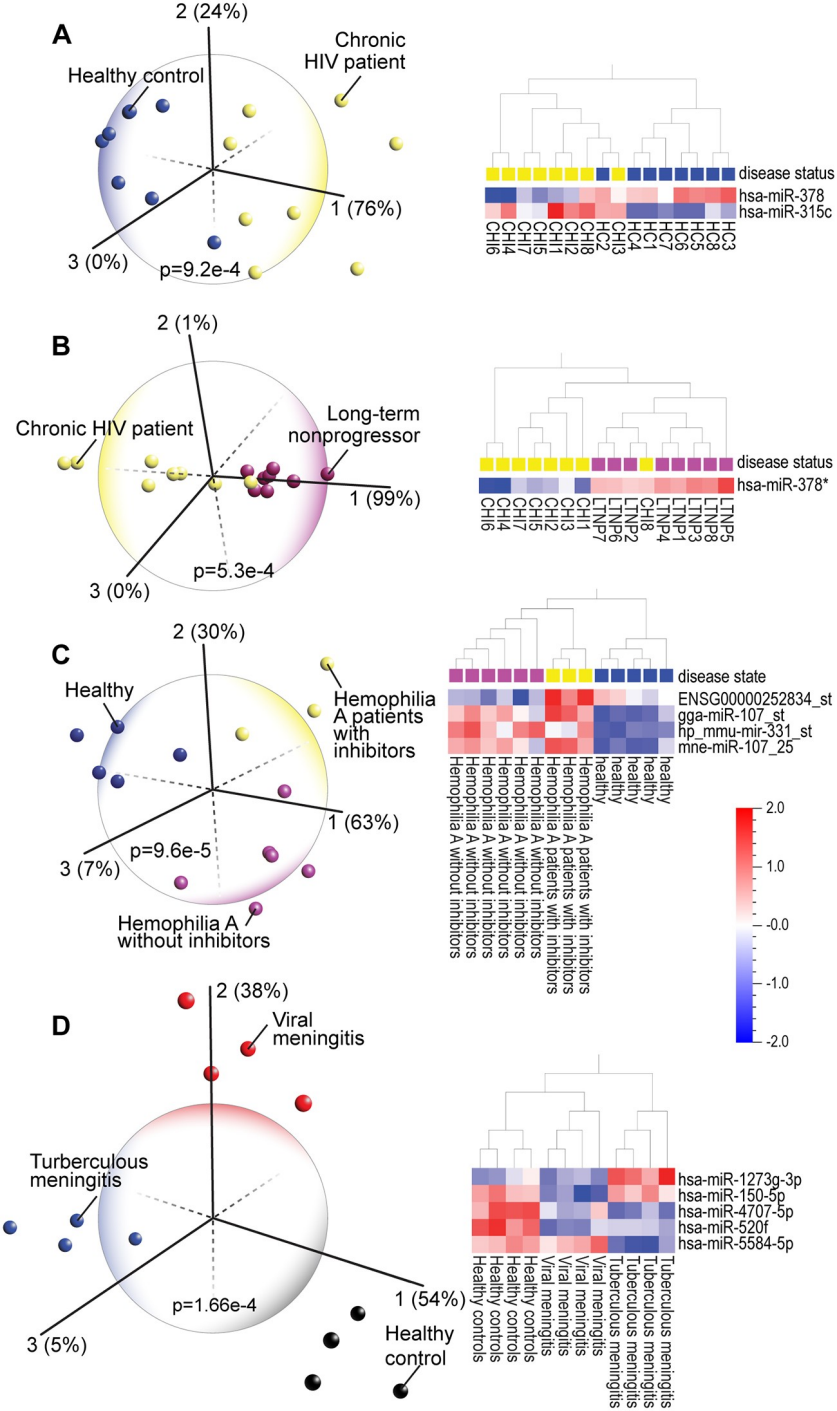
(A) Principal component analysis (PCA) and hierarchical clustering heatmap of blood monocyte miRNA expression profiles in chronic HIV (CHI), Long-term non-progressor (LTNP, HIV positive but asymptomatic) patients and healthy subjects (GSE38556). One miRNA, miR-378 (miR-378* is a lower level expressed form of the same miRNA) discriminates most of the HIV patients from controls and (B) also discriminates the majority of asymptomatic LTNP individuals from the chronic HIV group. (C) PCA and hierarchical clustering heatmap clearly distinguish hemophilia patients (GSE65581) from healthy controls and discriminates hemophilia A patients with inhibitors (development of neutralizing anti-FVIII antibodies) from those hemophilia A patients who did not develop inhibitors. (D) PCA and heatmap analysis aids in diagnosis of diseases, such as viral and tuberculous meningitis (GSE131708), that are difficult to differentiate clinically and identifies specific pro-survival miRNAs, i.e. miR-1273 that are associated with the less serious (viral) form of this disease.

Although HIV can be detected by a blood test, differentiating patients who will or will not progress to acquired immune deficiency syndrome (AIDS) would be helpful in planning and monitoring treatment. PCA and heatmap analyses of miRNA profiles of chronic HIV (CHI), Long-term Non-progressors (LTNP; infected individuals who have not progressed to AIDS) and healthy subjects differentiated these three groups (Fig 5A and 5B). Insight into disease resistance came from understanding the functional role of a single miRNA, miR-378*. This miRNA, which discriminates the majority of LTNPs from CHI patients and healthy subjects, targets the HIV envelope protein [69]. Moreover, miR-378* regulates glucose and lipid homeostasis by modulating hepatic insulin signaling [70] suggesting potential mechanisms for how LTNP can remain asymptomatic despite being infected with HIV. In these patients, miR-378* levels might be interfering with the utilization of metabolic substrates by the infecting virus.

PCA and heatmap analyses are also helpful in discriminating hemophilia A patients with endogenous inhibitors (neutralizing anti-FVIII antibodies) from those hemophilia A patients without inhibitors as well as clearly distinguishing both hemophilia A groups from healthy subjects (Fig 5C). Interestingly, miR-107 which distinguishes both types of hemophilia A patients from healthy subjects, is also known to be a potential biomarker of AD [71] and has been shown to mediate the effects of opioid and AD drugs [72, 73]. We found that PCA and heatmap analyses aids in diagnosis of diseases, such as viral and tuberculous meningitis, that are difficult to differentiate clinically (Fig 5D) [74]. We identified specific pro-survival miRNAs, such as miR-1273, that are associated with the less serious (viral) form of this disease. And miRNAs that identify both types of meningitis, such as miR-4707, are also implicated in other brain disorders [75] and cancer [76]. We further confirmed that the dysregulation of blood miRNAs associated with chronic inflammation distinguish the majority of patients with disorders such as sickle cell disease and chronic obstructive pulmonary disease (COPD) (S5 and S6 Figs). Across all the datasets we repeatedly observed that the functional roles of miRNAs, such as miR-182 in COPD, are consistent with their altered expression in each disease [77].

### PCA and heatmap analyses distinguished between similar nervous system disorders

**Fig 6:**
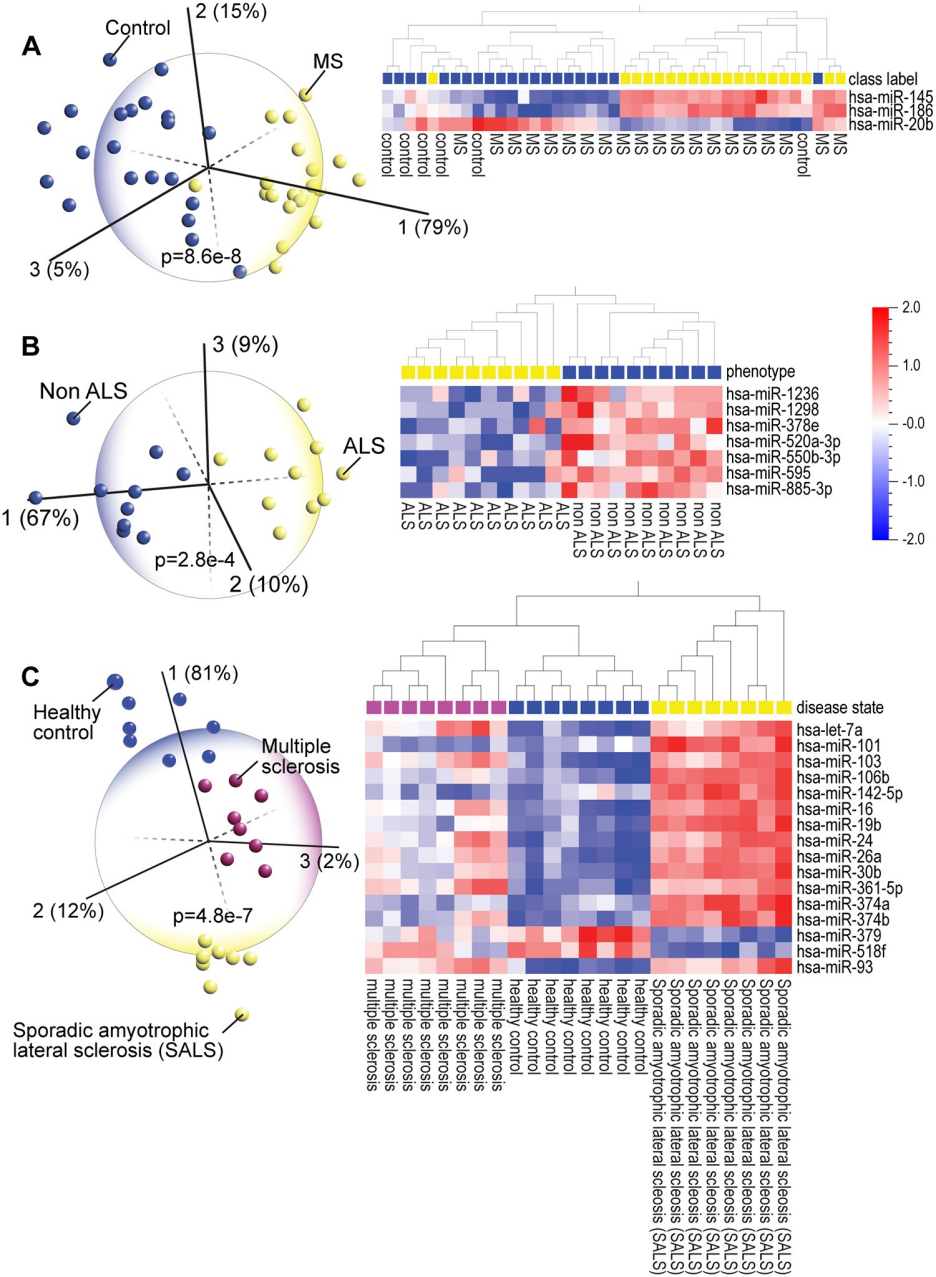
Principal component analysis (PCA) and hierarchical clustering heatmap of blood miRNA expression profiles in (A) multiple sclerosis patients [MS] (GSE17846), and spinal cord homogenate miRNA expression in (B) amyotrophic lateral sclerosis patients [ALS] (GSE52670) shows that PCA analysis of blood or tissue miRNA profiling has diagnostic potential for degenerative diseases. (C) PCA and hierarchical clustering analysis of blood monocyte miRNA expression in MS and sporadic ALS patients and healthy controls (GSE39643) shows that patients of these diseases, which in the early stages present with similar symptoms, can be clearly distinguished from each other as well as the control group. The discriminating miRNAs are prominently linked to inflammation and immune function.

In the early stages, both multiple sclerosis (MS) and amyotrophic lateral sclerosis (ALS) show similar symptoms such as muscle weakness and fatigue [78]. Early diagnosis of MS and ALS is complicated by similarities to other neurological disorders. Several studies report evidence for blood biomarkers for ALS [79], including miRNAs [80]. PCA enabled clear identification of the patients with each disease (Fig 6A and 6B, S7 Fig). We confirmed the published observation that miR-145 was the single best discriminating blood miRNA marker for patients with relapsing-remitting MS vs healthy subjects [81]. The upregulation of miR-145 is potentially a protective response [82] but its inhibition is also shown to be neuroprotective [83]. The surprising finding from our analysis is that two other miRNAs, miR-186 and miR-20b, that are associated with immune regulation, could also serve as discriminating markers of MS; miR-186 is involved in autoimmunity [84] and miR-20b, which inhibits inflammation, is downregulated in many MS patients [85]. Similar to MS, in PCA of ALS, we found that three upregulated miRNAs (miR-1236, miR-1298, miR-378), mitigate inflammation in healthy subjects [25, 86, 87]. In contrast, downregulation of miR-550 is associated with increased inflammation in ALS patients [88]. In other diseases such as atherosclerosis, inhibition of miR-103, which is downregulated in healthy controls, attenuates inflammation [89].

The underlying pathological mechanisms can be inferred through the analysis of a third dataset comparing healthy controls with MS and sporadic ALS patients (Fig 6C). Interestingly, all the miRNAs that identify sporadic ALS are expressed in the opposite direction in healthy subjects and show intermediate expression levels in MS patients. A distinct set of miRNAs involved in regulating immune function and cell death or survival were differentially expressed in all three groups supporting findings in previous genome-wide studies [90]. Both miR-101 and miR-30b contribute to inflammatory cytokine-meditated cell dysfunction [91], miR-374 [92] and miR-379 are also involved in MS pathology [93], and miR-361 is implicated in MS [94]. Since miR-93 has been shown to relieve inflammation [95], presumably by upregulating anti-inflammatory target genes, it is notable that it is expressed at low levels in only the healthy controls and it is highly expressed in both the MS and sporadic ALS patients. Although there is no cure for either disease, a blood test that accurately identified these patients, using these miRNAs as biomarkers, would improve quality of life for patients with these disorders since there are specific drugs that could alleviate symptoms of immune dysfunction (MS) [96] or help manage symptoms (ALS) [97].

### Blood miRNAs can identify neurodegenerative and other brain disorders

**Fig 7:**
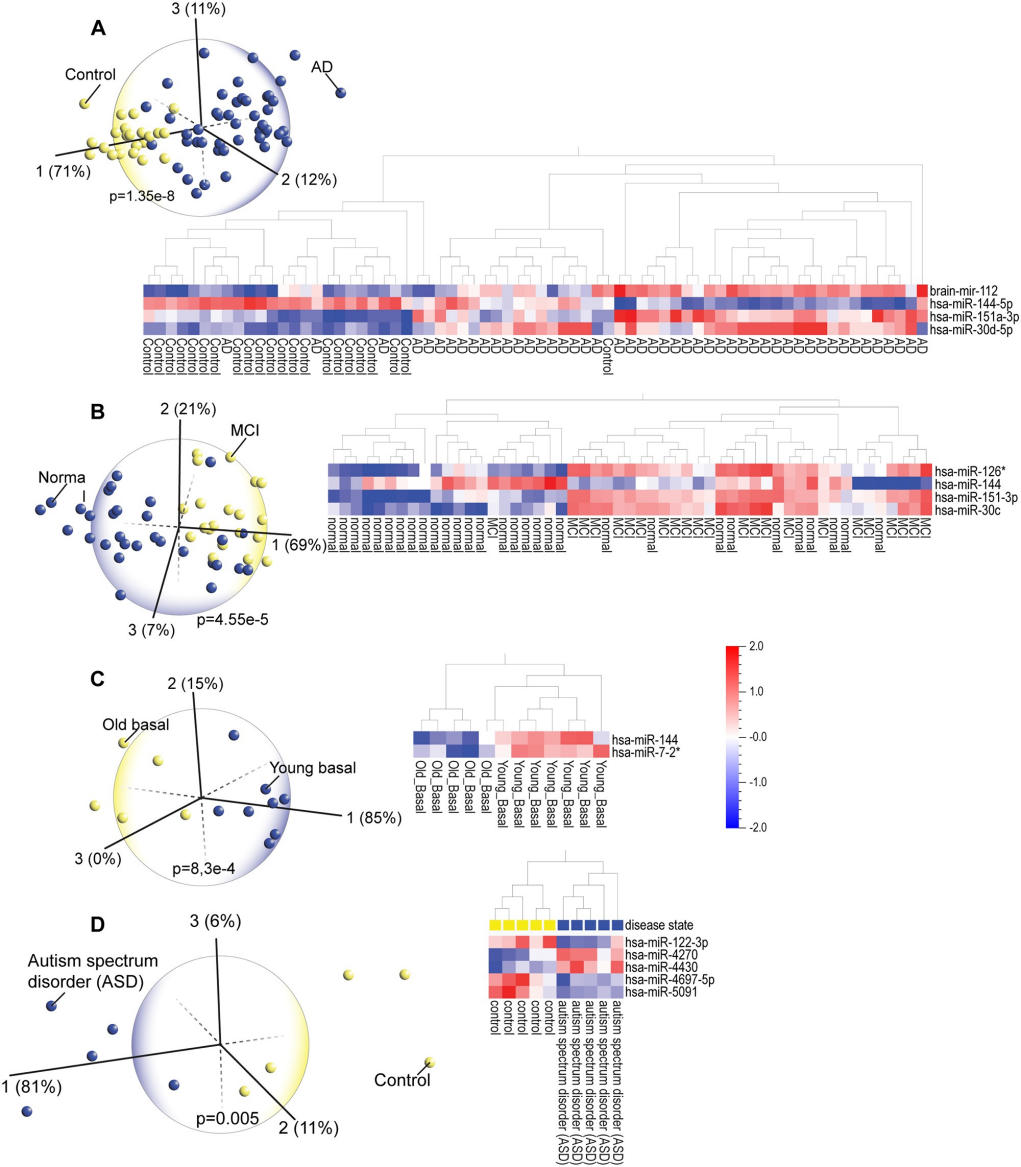
(A) Principal component analysis (PCA) and hierarchical clustering heatmap of blood miRNA expression profiles in Alzheimer’s disease (AD) patients vs healthy controls (GSE46579) shows that four discriminating miRNAs can identify the majority of the AD patients. (B) PCA and hierarchical clustering heatmap of blood plasma miRNA expression profiles in patients with mild cognitive impairment (MCI) (GSE90828) shows that most of the MCI patients can be identified via expression of four discriminating miRNAs. Note in both heatmaps that patterns of differential expression of three miRNAs (miR-144, miR-151 and miR-30) are very similar in the AD and MCI cohorts. (C) PCA and hierarchical clustering heatmap analysis of miRNA expression in aging and young muscle biopsies (GSE23527) shows that miR-144 is also a differentiating marker of aging. (D) PCA and hierarchical clustering heatmap analysis also facilitates the diagnosis of other brain disorders such as autism spectrum disorder (ASD); peripheral blood miRNA levels clearly distinguish ASD patients from healthy controls.

Studies report that distinct panels of plasma miRNAs may be biomarkers of MCI [98] and AD [99]. Altered levels of miRNAs were also found in the cerebrospinal fluid of patients diagnosed with young-onset AD [100]. PCA of blood miRNA datasets (Fig 7A and 7B) showed that three miRNAs, miR-144, miR-30 and miR-151, were expressed similarly in AD and MCI patients, suggesting common pathological mechanisms. MiR-144, decreased in most of the AD and many of the MCI patients, is associated with AD [101]. High levels of miR-30 have been linked to presenilin mutations in AD patients [102] and are high in both AD and MCI cohorts. The third miRNA, miR-151, is involved in memory processing (long-term potentiation), and is associated with AD [103]. The heatmap shows that levels of miR-151 are low in most normal subjects and high in subjects with AD or MCI. We observed that miR-144 and miR-30 are also dysregulated in patients with T2D (see Fig 3). Considering that cardiovascular disease, hypertension, stroke and diabetes are risk factors for AD and vascular dementia, miRNAs that are dysregulated in T2D patients as well as those with MCI and AD may reflect that risk. On the other hand, we also found that high levels of miR-144 may be a potential biomarker of healthy aging, because it is highly expressed in healthy aging subjects and differentiates young from old muscle (Fig 7C). Several subjects with the MCI miRNA signature were diagnosed as phenotypically normal, a known phenomenon, suggesting that these patients are disease resistant. Identifying unique features in this resistant population could determine which cell signaling pathways could be therapeutically targeted to treat MCI and AD. On a final note, another brain disorder, autism spectrum disorder (ASD) is difficult to diagnose due to the heterogeneity of ASD [104] but PCA and heatmap analyses clearly differentiated those with ASD (Fig 7D).

The common thread among these brain disorders is inflammation and dysregulated immune responses [105–107], which have a causal role in AD and other dementias [108, 109]. Understanding these mechanisms has already proven beneficial; for example, diabetes drugs that have anti-inflammatory properties protect against AD [55]. Demographic characteristics and analysis results of GEO datasets are summarized in Table 1.

**Table 1:** 

Fig. Nbr.	Disease/Disorder	Gene Expression Omnibus accession number	p-value	q-value^‡^	Nbr. of patients: healthy controls (P:C)	Sample	Discriminating miRNA variables (hsa-miR-?)
1A	Acute myocardial infarction	GSE24548	0.0047	0.037	4P^†^:3C^†^	Platelets	101, 106b, 140-5p, 142-3p, 142-5p, 15a*, 17*, 199a-5p, 19a, 200a, 20a*, 20b, 21, 21*, 219-5p, 223, 24–1*, 26b, 29b, 29c, 301a, 30a*, 30e, 32, 324-5p, 326, 33a, 33b, 340, 342-3p, 34a, 421, 454, 545, 548a-5p, 590-5p, 598
1B	Cardiac arrest	GSE34643	3.90E-04	0.07	5P^†^:5C^†^	Plasma	574-5p, 1914*, miRBrightCorner30
1C	Premature coronary artery disease	GSE28858	8.00E-06	7.30E-04	12P:12C	Platelets	548g, 1247, 1236, 526b, 551b*, 1278
2A	Intracerebral hemorrhage	GSE43618	0.0024	n/d	4P^†^:2C^†^	Plasma	1228, 215, 516a-5p
2B	High blood pressure	GSE28344	5.80E-04	0.29	5P:3C	Renal medulla	208b
2C	Sepsis-induced acute kidney injury	GSE94717	6.50E-04	0.02	12P:3C	Venous blood	195-5p, 449c-5p, 4299, 3181, 3689f, 4704-5p, 21-3p, 188-3p, 1292-5p
3A	Type 1 diabetes	GSE55099	0.002	0.2	12P:10C	Peripheral blood mononuclear cells	1225-3p, 16, 26a,30a, 320a, 320b, 617
3B	Type 2 diabetes	GSE21321	1.48E-04	0.04	16P:10C	Whole blood	144 (4 probes), 30d (4 probes)
4A	Colon cancer	GSE16512	4.35E-05	8.20E-04	9P:14C	Serum	197, 328, 885-5p
4B	Prostate cancer	GSE16512	5.40E-06	4.20E-04	6P:14C	Serum	197, 328, 636, 885-5p, 92a
4C	Chronic lymphocytic leukemia (proliferating vs quiescent)	GSE53270	3.45E-04		4 Proliferating: 4 Quiescent	Whole blood	150, 720
4D	Diffuse large B-cell lymphoma(DLBCL) / Burkitt lymphoma(BL)	GSE22420	7.00E-25	1.58E-23	86DLBCL:64BL	Tumor cells	miRNA list available in PMID: 21701491
4E	Bladder cancer	GSE113486	1.00E-139	5.60E-139	392P:100C	Serum	miRNA list available in PMID: 30382619
5A	Chronic HIV patient	GSE38556	9.20E-04	0.1	8P:8C	Blood monocytes	378. 315c
5B	Chronic HIV patient / Long-term nonprogressor	GSE38556	5.30E-04	0.1	8HIV:8LTNP	Blood monocytes	378*
5C	Hemophilia A w/ and w/o inhibitors	GSE65581	9.60E-05	n/d	9P:5C	Whole blood	Multispecies miRNAs: ENSG00000252834_st gga-miR-107_st hp_mmu-mir-331_st mne-miR-107_25
5D	Viral meningitis / tuberculous meningitis	GSE131708	1.66E-04	0.06	8P:4C	Peripheral blood mononuclear cells	1273g-3p, 150-5p, 4707-5p, 520f, 5584-5p
6A	Multiple sclerosis (MS)	GSE17846	8.60E-08	1.60E-05	20P:21C (partial heatmap shown)	Whole blood	145, 186, 20b
6B	Amyotrophic lateral sclerosis (ALS)	GSE52670	2.80E-04	0.026	10P:10C	Spinal cord homogenate (post-mortem)	1236, 1298, 378e, 520a-3p, 550b-3p, 595, 885-3p
6C	MS vs ALS vs healthy control	GSE39643	4.80E-07	4.40E-06	16P:8C	Blood monocytes	7a, 101, 103, 106b, 142-5p, 16, 19b, 24, 26a, 30b, 361-5p, 374a, 374b, 379, 518f, 93
7A	Alzheimer’s disease	GSE46579	1.35E-08	1.65E-06	48P:22C (partial heatmap shown)	Whole blood	112, 144-5p, 151a-3p, 30d-5p
7B	Mild cognitive impairment	GSE90828	4.55E-05	0.007	23P:30C (partial heatmap shown)	Plasma	126*, 144, 151-3p, 30c
7C	Old / young muscle	GSE23527	8.30E-04	0.22	17 old:19 young (partial heatmap shown)	Skeletal muscle	144, 7–2*
7D	Autism spectrum disorder (ASD)	GSE67979	0.005	n/d	5P:5C	Whole blood	122-3p, 4270, 4430, 4697-5p, 5091

n/d = no data.

^‡^ False discovery rate.

^†^ pooled samples.

## Discussion

Using PCA, a powerful data reduction method, we characterized patient cohorts from downloaded GEO datasets of peripheral blood miRNA representing a broad spectrum of human diseases. PCA, in all its variations (e.g. Factor Analysis, Singular Value Decomposition, Singular Spectrum Analysis) is considered an unbiased, hypothesis-generating tool because it creates a statistical mechanistic platform for modeling biological changes without strong *a priori* assumptions [110]. Investigating the published literature on the discriminating miRNA variables from the hierarchical clustering heatmaps for each disease, showed that the miRNAs have functional roles relevant to the pathophysiology of each disease. This survey of PCA and heatmaps of blood/ biofluid miRNA datasets provided four key insights.

First, a universal blood test is possible. Since circulating miRNAs are dysregulated across a diverse spectrum of diseases, a universal blood test from a routine blood sample is a realistic goal. The ability to measure the changes in expression of miRNAs linked to specific diseases would provide a new diagnostic tool. For a universal blood test to be effectively utilized in clinical settings, it would be important to construct a comprehensive reference database of PCA plots and heatmaps representing the entire spectrum of known human diseases. The present analysis suggests that this is an attainable goal.

Second, the functional roles of the disease-discriminating miRNAs validated our findings. Inflammation surfaced as a key mechanistic underpinning of multiple chronic diseases. Published evidence supports the central role of chronic inflammation in heart disease, diabetes and AD, and also as a contributing factor to MS and ALS [111]. Additionally, in all datasets examined, literature searches showed that the functional, posited role of discriminating miRNAs correlated with their expression in patients or healthy controls. For instance, in the sepsis-induced AKI dataset, miR-195, which inhibits inflammation [112] is highly upregulated only in the healthy control group.

For some diseases, earlier diagnosis could be life changing because there are drug therapies that could improve the quality and duration of life, such as riluzole (Rilutek, S1 Reference) for MS and ALS. Furthermore, the finding that chronic inflammation is the common underlying mechanism of many diseases has important implications. For example, a recent study by Lavin et al, showed that although a pro-inflammatory blood and muscle profile is associated with aging, life-long exercise positively impacted muscle heath in aging by promotion of anti-inflammatory gene and protein expression in skeletal muscle [113].

Third, miRNA changes shared by divergent diseases indicate a mechanistic link or common underlying pathology that could be therapeutically addressed with common drugs; for example, a common set of miRNAs, including miR-144, are found to be dysregulated in diabetes, MCI and AD. Two miRNAs, miR-144 and miR-30, which discriminate among the IFG, T2D and control groups, are the same miRNAs that identify the AD and MCI groups, suggesting that these miRNAs may be linked to the common finding of dementia and cognitive dysfunction in these diseases. These distinctly different diseases may be treated by one drug, for example, since T2D patients are at increased risk of AD and vascular dementia, it is notable that metformin–a glucose-lowering drug–protects against AD [55]. Since modification of diet and lifestyle have reduced or delayed onset of AD (S1 Reference), earlier diagnosis of MCI, the first stage of dementia, could prompt similar interventions. Changes in miRNA expression levels could also serve as biomarkers of effective responses to treatments for these diseases.

Fourth, current evidence supports using disease-altered miRNA panels as predictive and diagnostic markers of heart disease [114], MCI [115] and AD [116]. PCA of serum miRNA expression was recently shown to predict dementia in AD patients [117]. Although our objective was not to identify and validate miRNA biomarkers, it is evident that, in future translational studies, the discriminating miRNAs that result from PCA and heatmap analysis of these studies may prove to be robust biomarkers of each of these diseases. In addition, recent studies showed that blood miRNAs can serve as potential biomarkers of complex psychiatric disorders such as schizophrenia [118]. The identification of potential blood biomarkers via PCA and heatmap analyses could facilitate a computational biology approach to drug discovery for neurodegenerative disorders [119]. In addition, some of the miRNA markers found in AD and MCI are also dysregulated in aging muscle [120]. This supports previous reports that peripheral blood miRNAs can serve as biomarkers of normal aging as well as age-related diseases [121]. The idea of using publicly available data of miRNA-seq profiles for diagnosis of AD has been recently proposed [116].

On a cautionary note, this analysis did not permit estimates of sensitivity, specificity or positive or negative predictive value of these PCA and heatmap analyses. We also observed that the discriminating miRNA variable lists were often different from published analyses of the data [122]. This could be attributed to differences in statistical and machine learning algorithms used in the analysis of miRNA datasets by different investigators. Because many of these datasets were lacking a peer-reviewed publication, we could not compile a comprehensive comparison of our discriminating miRNA lists with the ones in each GEO submission. However, our PCA analysis was clear and unequivocal in identifying the patient populations in all these GEO datasets. Using the same data reduction algorithm, we correctly identified those patients previously identified by different diagnostic methods specific for each disease. With currently available *in silico* target prediction algorithms, the biological significance (i.e., disease mechanisms), of the discriminating miRNA variables is not always clear [80]. However, as we have shown, in most of these PCA/heatmap sets, we found an association of some of the discriminating miRNAs with known disease mechanisms; for instance, the increased expression of miR-378*, which inhibits production of the HIV envelope protein and viral replication in HIV infected individuals who do not show disease symptoms.

There is ongoing progress in defining whole transcriptome blood miRNA profiles (miRNome) of human diseases; Keller et al., showed consistently deregulated miRNA profiles for a broad spectrum of 14 human diseases [123]. This will result in a massive accumulation of blood profiling data that can be interrogated for diagnostic purposes. Given the concordance of the public data with our PCA analysis, we suggest that a searchable database of PCA and heatmap analyses of blood miRNA expression data, obtained from a variety of platforms, could be used together with other evidence-based measures to identify patients with specific diseases and facilitate personalized medicine.

## Supporting information

S1 Table Summary of principal component analysis (PCA) workflow in Qlucore Omics Explorer. (PDF)

S1 Fig Principal component analysis and hierarchical clustering heatmap of miRNA expression in pooled blood plasma miRNA expression profiles clearly distinguish patients with unstable angina pectoris from healthy subjects (GSE94605). (PDF)

S2 Fig Principal component analysis (PCA) and hierarchical clustering heatmap of miRNA expression in tissues of Burkitt lymphoma and Diffuse large B-cell lymphoma patients (GSE22420) shows that PCA of miRNA expression data in disease-associated tissues (if available from patient populations) can also facilitate identification of the patient cohort despite the complexity of the miRNA dataset which is reflected in the hierarchical clustering heatmap. (PDF)

S3 Fig Principal component analysis of serum miRNA expression clearly distinguishes 392 bladder cancer patients from 100 non-cancer controls (GSE113486). (PDF)

S4 Fig Peripheral blood miRNA profiles of 14 different diseases (GSE31568) were analyzed via microarray analysis by Keller et al., 2011 (Nature Methods). In all diseases, blood miRNA profiles were found dysregulated. For example, principal component analysis and hierarchical clustering heatmap analysis clearly identified prostate cancer patients from healthy controls.
(PDF)

S5 Fig Principal component analysis and hierarchical clustering heatmap of miRNA expression in circulating platelets from sickle cell disease patients (SCD) and control subjects (GSE41574) shows that only three discriminating miRNAs can identify the majority of the SCD patients. (PDF)

S6 Fig Principal component analysis and hierarchical clustering heatmap analysis shows that four blood miRNAs, associated with inflammation, help identify the majority of chronic obstructive pulmonary disease [COPD] patients from healthy controls (GSE31568). (PDF)

S7 Fig Principal component analysis and hierarchical clustering heatmap analysis of multiple sclerosis (MS) blood miRNA expression profiles (GSE31568) shows that three circulating miRNAs, associated with inflammation and immune function, can identify the majority of MS patients. (PDF)

S1 Reference (PDF)
