# Supplementary material for: Principal component analysis of blood microRNA datasets facilitates diagnosis of diverse diseases
Source: PLoS One. 2020 Jun 5;15(6):e0234185. doi: 10.1371/journal.pone.0234185 (PMC7274418; doi:10.1371/journal.pone.0234185)
Supplement: S1 Table — (PDF) [file pone.0234185.s001.pdf]

S1 Table. Summary of principal component analysis (PCA) work- low in Qlucore Omics Explorer.

1	Search NCBI GEO Datasets for a specific disease; obtain the GEO accession number.
2	Open QOE
3	(click) File
4	(click) Download
5	(click) GEO Dataset
6	Enter GSE number
7	[OK]
8	Download annotations if they exist → [OK]
9	Data loads and is displayed as a PCA plot
10	(click) Data tab
11	Log 2 transform
12	(click) Statistics box
13	(click) Perform variance filtering
14	Perform statistics filtering (e.g., two-group comparison or multigroup comparison)
15	Adjust p value and q value to desired significance
16	Visualize PCA plot and heatmap
17	Export data and images

Note: Steps 15 and 16 are interactive and simultaneous. PCA and heatmaps change according to p-value adjustments.
