# Supplementary material for: Principal component analysis of blood microRNA datasets facilitates diagnosis of diverse diseases
Source: PLoS One. 2020 Jun 5;15(6):e0234185. doi: 10.1371/journal.pone.0234185 (PMC7274418; doi:10.1371/journal.pone.0234185)
Supplement: S1 Fig — (PDF) [file pone.0234185.s002.pdf]

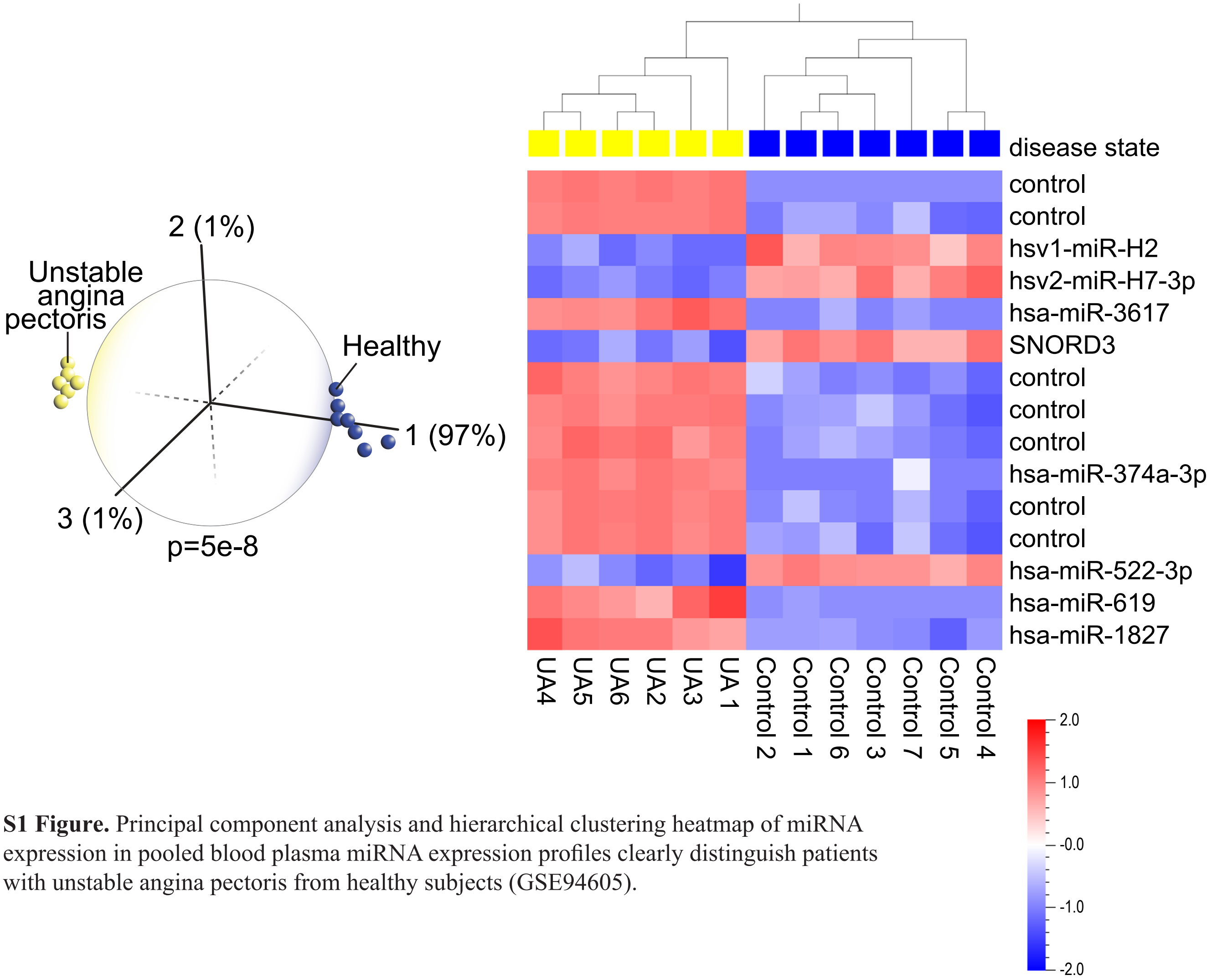

S1 Figure. Principal component analysis and hierarchical clustering heatmap of miRNA expression in pooled blood plasma miRNA expression profiles clearly distinguish patients with unstable angina pectoris from healthy subjects (GSE94605).
