# Supplementary figures and images for: Principal component analysis of blood microRNA datasets facilitates diagnosis of diverse diseases
Source: PLoS One. 2020 Jun 5;15(6):e0234185. doi: 10.1371/journal.pone.0234185 (PMC7274418; doi:10.1371/journal.pone.0234185)

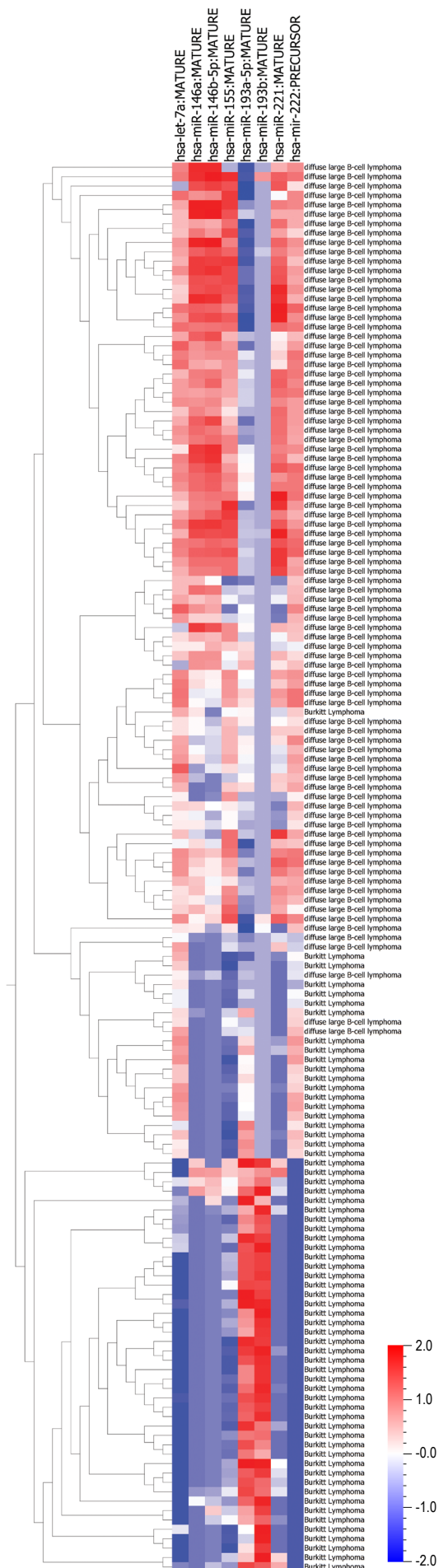[illegible]

Supplement: S2 Fig — (PDF) [file pone.0234185.s003.pdf]

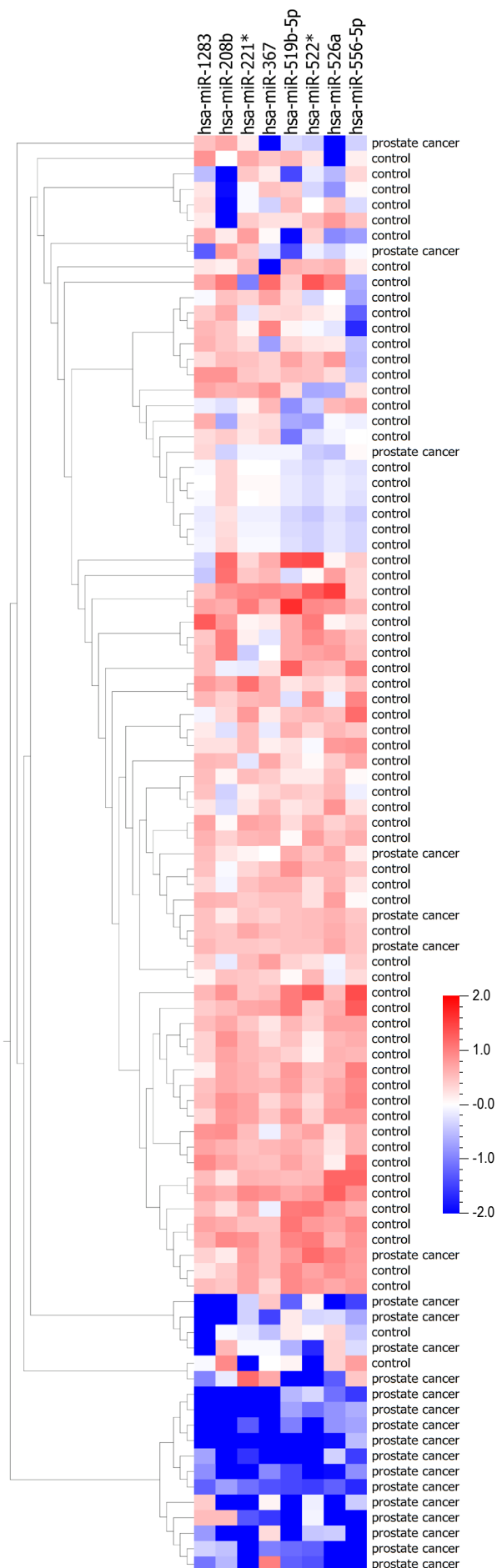

Supplement: S4 Fig — In all diseases, blood miRNA profiles were found dysregulated. For example, principal component analysis and hierarchical clustering heatmap analysis clearly identified prostate cancer patients from healthy controls. (PDF) [file pone.0234185.s005.pdf]

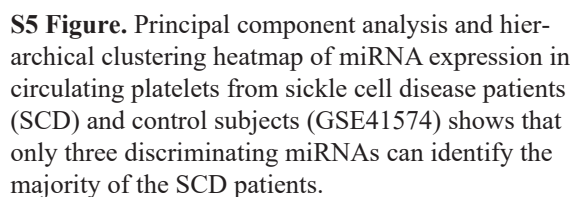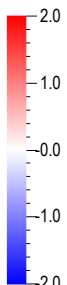

Supplement: S5 Fig — (PDF) [file pone.0234185.s006.pdf]
