# Supplementary material for: Principal component analysis of blood microRNA datasets facilitates diagnosis of diverse diseases
Source: PLoS One. 2020 Jun 5;15(6):e0234185. doi: 10.1371/journal.pone.0234185 (PMC7274418; doi:10.1371/journal.pone.0234185)
Supplement: S3 Fig — (PDF) [file pone.0234185.s004.pdf]

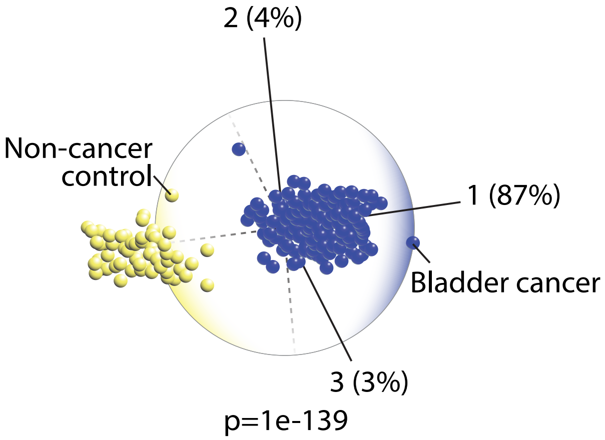

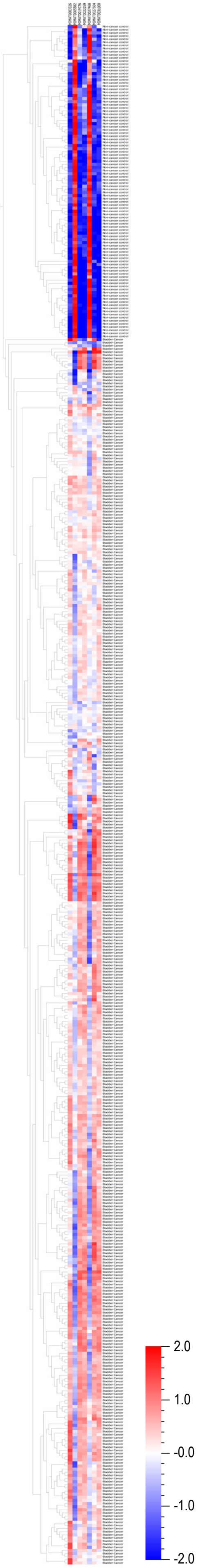

S3 Figure. Principal component analysis of serum miRNA expression clearly distinguishes 392 bladder cancer pa-tients from 100 non-cancer controls (GSE113486).
