# Supplementary material for: Principal component analysis of blood microRNA datasets facilitates diagnosis of diverse diseases
Source: PLoS One. 2020 Jun 5;15(6):e0234185. doi: 10.1371/journal.pone.0234185 (PMC7274418; doi:10.1371/journal.pone.0234185)

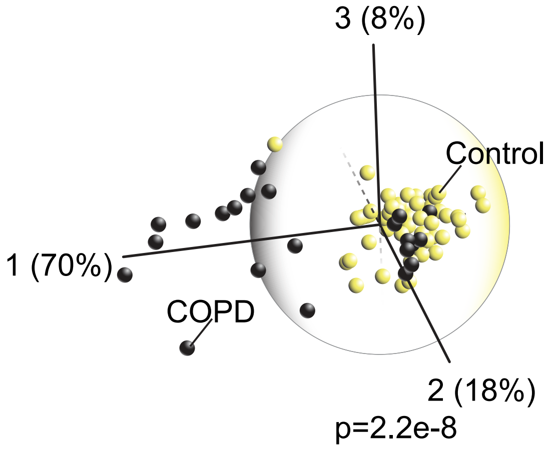

S6 Figure. Principal component analysis and hierarchical clustering heatmap analysis shows that four blood miRNAs, associated with inflammation, help identify the majority of chronic obstructive pulmonary disease [COPD] patients from healthy controls (GSE31568).

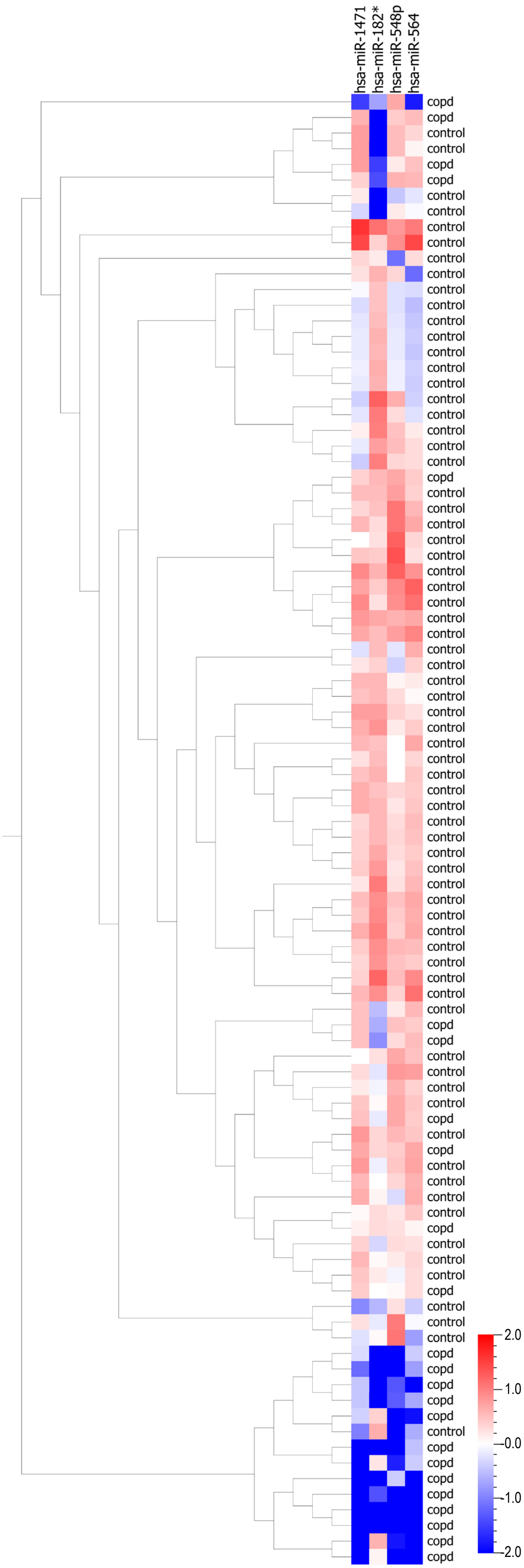

Supplement: S6 Fig — (PDF) [file pone.0234185.s007.pdf]
