# Supplementary material for: Principal component analysis of blood microRNA datasets facilitates diagnosis of diverse diseases
Source: PLoS One. 2020 Jun 5;15(6):e0234185. doi: 10.1371/journal.pone.0234185 (PMC7274418; doi:10.1371/journal.pone.0234185)
Supplement: S7 Fig — (PDF) [file pone.0234185.s008.pdf]

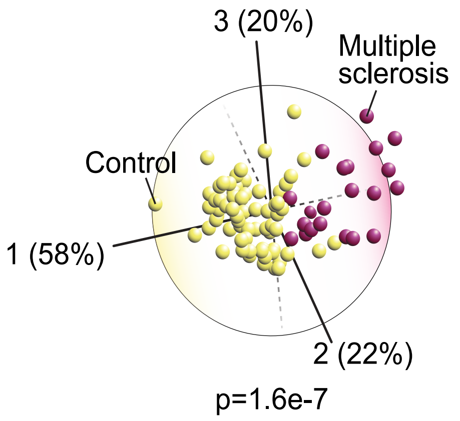

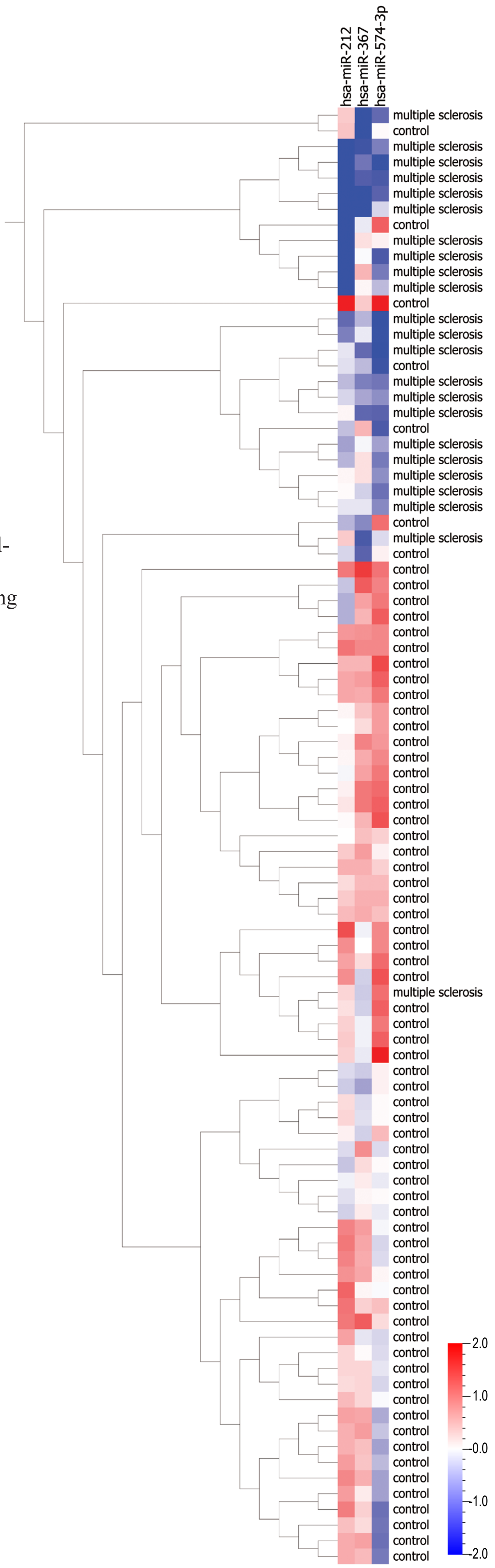

S7 Figure. Principal component analysis and hierarchical clustering heatmap analysis of multiple sclerosis (MS) blood miRNA expression profiles (GSE31568) shows that three circulating miRNAs, associated with inflammation and immune function, can identify the majority of MS patients.
