# Supplementary material for: Principal component analysis of blood microRNA datasets facilitates diagnosis of diverse diseases
Source: PLoS One. 2020 Jun 5;15(6):e0234185. doi: 10.1371/journal.pone.0234185 (PMC7274418; doi:10.1371/journal.pone.0234185)
Supplement: S1 Reference — (PDF) [file pone.0234185.s009.pdf]

References

Lifestyle modifications that improve outcome in human diseases.

1. Doughty KN, Del Pilar NX, Audette A, Katz DL. Lifestyle Medicine and the Management of Cardiovascular Disease. Curr Cardiol Rep. 2017;19(11):116. Epub 2017/10/06. doi: 10.1007/s11886-017-0925-z. PubMed PMID: 28980137.
2. Van Buren DJ, Tibbs TL. Lifestyle interventions to reduce diabetes and cardiovascular disease risk among children. Curr Diab Rep. 2014;14(12):557. Epub 2014/10/27. doi: 10.1007/s11892-014-0557-2. PubMed PMID: 25344792; PubMed Central PMCID: PMCPMC4209242.
3. Alkhatib A, Tsang C, Tiss A, Bahorun T, Arefanian H, Barake R, et al. Functional Foods and Lifestyle Approaches for Diabetes Prevention and Management. Nutrients. 2017;9(12). Epub 2017/12/02. doi: 10.3390/nu9121310. PubMed PMID: 29194424; PubMed Central PMCID: PMCPMC5748760.
4. Hill VA, Towfighi A. Modifiable Risk Factors for Stroke and Strategies for Stroke Prevention. Semin Neurol. 2017;37(3):237-58. Epub 2017/08/02. doi: 10.1055/s-0037-1603685. PubMed PMID: 28759906.
5. Samadian F, Dalili N, Jamalian A. Lifestyle Modifications to Prevent and Control Hypertension. Iran J Kidney Dis. 2016;10(5):237-63. Epub 2016/10/11. PubMed PMID: 27721223.
6. Fitch KV. Contemporary Lifestyle Modification Interventions to Improve Metabolic Comorbidities in HIV. Curr HIV/AIDS Rep. 2019. Epub 2019/11/30. doi: 10.1007/s11904-019-00467-0. PubMed PMID: 31776973.
7. Kolak A, Kaminska M, Sygit K, Budny A, Surdyka D, Kukielka-Budny B, et al. Primary and secondary prevention of breast cancer. Ann Agric Environ Med. 2017;24(4):549-53. Epub 2017/12/30. doi: 10.26444/aaem/75943. PubMed PMID: 29284222.
8. Avancini A, Sartori G, Gkountakos A, Casali M, Trestini I, Tregnago D, et al. Physical Activity and Exercise in Lung Cancer Care: Will Promises Be Fulfilled? Oncologist. 2019. Epub 2019/11/28. doi: 10.1634/theoncologist.2019-0463. PubMed PMID: 31771988.
9. Zhou J, Zhang Z, Zhou H, Qian G. Diabetic Cognitive Dysfunction: From Bench to Clinic. Curr Med Chem. 2019. Epub 2019/02/08. doi: 10.2174/1871530319666190206225635. PubMed PMID: 30727866.
10. Blumenthal JA, Smith PJ, Mabe S, Hinderliter A, Welsh-Bohmer K, Browndyke JN, et al. Longer Term Effects of Diet and Exercise on Neurocognition: 1-Year Follow-up of the ENLIGHTEN Trial. J Am Geriatr Soc. 2019. Epub 2019/11/23. doi: 10.1111/jgs.16252. PubMed PMID: 31755550.
11. Kivipelto M, Mangialasche F, Ngandu T. Lifestyle interventions to prevent cognitive impairment, dementia and Alzheimer disease. Nat Rev Neurol. 2018. Epub 2018/10/07. doi: 10.1038/s41582-018-0070-3. PubMed PMID: 30291317.
12. Tsai CL, Pai MC, Ukropec J, Ukropcova B. Distinctive Effects of Aerobic and Resistance Exercise Modes on Neurocognitive and Biochemical Changes in Individuals with Mild Cognitive Impairment. Curr Alzheimer Res. 2019;16(4):316-32. Epub 2019/03/02. doi: 10.2174/1567205016666190228125429. PubMed PMID: 30819077.
13. Scarmeas N, Anastasiou CA, Yannakoulia M. Nutrition and prevention of cognitive impairment. Lancet Neurol. 2018;17(11):1006-15. Epub 2018/09/25. doi: 10.1016/S1474-4422(18)30338-7. PubMed PMID: 30244829.

Riluzole and lifestyle modifications for ALS and MS

14. Miller RG, Mitchell JD, Moore DH. Riluzole for amyotrophic lateral sclerosis (ALS)/motor neuron disease (MND). Cochrane Database Syst Rev. 2012;(3):CD001447. Epub 2012/03/16. doi: 10.1002/14651858.CD001447.pub3. PubMed PMID: 22419278.
15. Connick P, De Angelis F, Parker RA, Plantone D, Doshi A, John N, et al. Multiple Sclerosis-Secondary Progressive Multi-Arm Randomisation Trial (MS-SMART): a multiarm phase IIb randomised, double-blind, placebo-controlled clinical trial comparing the efficacy of three neuroprotective drugs in secondary progressive multiple sclerosis. BMJ Open. 2018;8(8):e021944. Epub 2018/09/01. doi: 10.1136/bmjopen-2018-021944. PubMed PMID: 30166303; PubMed Central PMCID: PMCPMC6119433.
16. Johansson S, Ytterberg C, Gottberg K, Holmqvist LW, von Koch L, Conradsson D. Participation in social/lifestyle activities in people with multiple sclerosis: Changes across 10 years and predictors of sustained participation. Mult Scler. 2019:1352458519881991. Epub 2019/10/18. doi: 10.1177/1352458519881991. PubMed PMID: 31621488.
17. Riccio P, Rossano R. Nutrition facts in multiple sclerosis. ASN Neuro. 2015;7(1). Epub 2015/02/20. doi: 10.1177/1759091414568185. PubMed PMID: 25694551; PubMed Central PMCID: PMCPMC4342365.
18. van Groenestijn AC, van de Port IG, Schroder CD, Post MW, Grupstra HF, Kruitwagen ET, et al. Effects of aerobic exercise therapy and cognitive behavioural therapy on functioning and quality of life in amyotrophic lateral sclerosis: protocol of the FACTS-2-ALS trial. BMC Neurol. 2011;11:70. Epub 2011/06/16. doi: 10.1186/1471-2377-11-70. PubMed PMID: 21672211; PubMed Central PMCID: PMCPMC3125309.
